# Computed tomography findings after radiofrequency ablation in locally advanced pancreatic cancer

**DOI:** 10.1007/s00261-018-1519-y

**Published:** 2018-02-28

**Authors:** Steffi J. E. Rombouts, Tyche C. Derksen, Chung Y. Nio, Richard van Hillegersberg, Hjalmar C. van Santvoort, Marieke S. Walma, Izaak Q. Molenaar, Maarten S. van Leeuwen

**Affiliations:** 1 Department of Surgery, University Medical Center Utrecht Cancer Center, 3508 GA Utrecht, PO Box 85500, The Netherlands; 2 Department of Radiology, Academic Medical Center Amsterdam, PO Box 22660, 1100 DD Amsterdam, The Netherlands; 3 Department of Surgery, Sint Antonius Hospital, Nieuwegein, The Netherlands; 4 Department of Radiology, University Medical Center Utrecht Cancer Center, 3508 GA Utrecht, PO Box 85500, The Netherlands

**Keywords:** Radiofrequency ablation, Computed tomography, Locally advanced pancreatic cancer, Imaging findings

## Abstract

**Purpose:**

The purpose of the study was to provide a systematic evaluation of the computed tomography(CT) findings after radiofrequency ablation (RFA) in locally advanced pancreatic cancer(LAPC).

**Methods:**

Eighteen patients with intra-operative RFA-treated LAPC were included in a prospective case series. All CT-scans performed prior to RFA and 1 week and 3 months of post-RFA, according to standard regimen, were assessed by two radiologists in consensus, using standardized radiological scoring lists.

**Results:**

51 CT-scans were assessed. One week after RFA, the ablation zone was visible in all patients as a (partially) sharply defined (83%), heterogeneous area (94%). At 3 months of follow-up, the ablation zone was completely invaded by tumor in 67% of patients and still present, but decreased in 33%. In two patients (11%), local thrombosis and/or occlusion of the superior mesenteric vein occurred. The occlusions persisted without clinical consequences and the thrombosis disappeared. A peripancreatic fluid collection was visible 1 week after RFA in 3 patients, wherein the ablation zone extended ventrally outside of the pancreas.

**Conclusions:**

Directly after RFA for LAPC, a well-defined ablation zone is visible on CT-imaging. This ablation zone is usually replaced by tumor ingrowth after 3 months. Moreover, the ablation zone regularly included vascular structures, with rare asymptomatic venous occlusion or thrombosis and without adverse effects on arteries.

**Electronic supplementary material:**

The online version of this article (10.1007/s00261-018-1519-y) contains supplementary material, which is available to authorized users.

Pancreatic cancer is the twelfth most common cancer worldwide, with an incidence of 338.000 patients annually and the fifth leading cause of cancer-related death with an overall five-year survival rate of 6%, reflecting its poor prognosis [1] .

The only hope for cure is surgical resection, which is possible in only 20% of patients. At presentation, 40%–50% have distant metastases and another 30%–40% present with locally advanced pancreatic carcinoma (LAPC), without metastatic spread [2, 3]. For patients with LAPC, chemotherapy has been standard treatment for over a decade, providing only a marginal survival benefit [3]. In recent years, radiofrequency ablation (RFA), a local ablative therapy, has been explored as new treatment option to prolong survival substantially. RFA involves the implantation of one or more electrodes directly into the tumor. These needles produce a high frequency alternating current, which leads to frictional heating and thus tissue destruction by thermal coagulation and protein denaturation. Several studies have demonstrated RFA to be feasible and safe in LAPC, with promising survival benefits up to 25.6 months [4].

To interpret the radiological effects of RFA, such as the ablation success and detection of recurrence, familiarity with imaging directly after the procedure and during follow-up is essential. According to a recent published overview regarding imaging evaluation of pancreatic cancer, computed tomography (CT) is the preferred imaging modality for assessing pancreatic adenocarcinoma [5]. However, studies systematically describing these CT-findings after RFA in LAPC are lacking [6–9]. This lack gives rise to numerous questions, such as: what is the appearance of the ablation zone shortly after RFA and what changes occur in the ablation zone over time? What are the effects on vascular structures involved in or nearby to the ablation zone? And how does the tumor progress during follow-up after RFA?

Therefore, the purpose of this study is to provide a systematic evaluation of the CT-findings after RFA in patients with LAPC, by describing the changing characteristics over time of the tumor, ablation zone, and their relation to surrounding vessels during follow-up.

## Materials and methods

### Patient selection

At our institution, between November 2012 and April 2014, 18 patients with histologically proven LAPC were treated with RFA intra-operatively. Patients were selected after multidisciplinary board discussion, consisting of surgeons, radiologists, and oncologists. For the definition of LAPC, the guidelines of the Dutch Pancreatic Cancer Group were used: superior mesenteric artery (SMA), celiac trunk or common hepatic artery (CHA) involvement exceeds 90° and/or involvement of the portal vein (PV) and/or superior mesenteric vein (SMV) exceeds 270°, without metastases [10, 11]. Other selection criteria included the following: technically suitable for radiofrequency ablation as judged by the interventional radiologists; no distant metastasis and the possibility for follow-up within the treating hospital. As patients were selected from multiple originating hospitals, pre-RFA patient treatment varied in concordance with local guidelines and the treating oncologist’s preference.

This case series was part of the feasibility study in preparation of a larger clinical trial, which is currently underway as a multicenter randomized controlled trial in the Netherlands (The PELICAN Trial). Institutional Review Board approval and patient informed consent were acquired.

### RFA procedure

All procedures were performed under general anesthesia, by one dedicated interventional radiologist.

After explorative laparotomy, a Kocher manoeuvre and division of the gastrocolic ligament was performed to expose the pancreas. Ultrasound was used to assess the interventional strategy. The intention of RFA treatment in LAPC is essentially a form of tumor debulking rather than total tumor ablation. Therefore, a minimal safety margin between the RFA needle and surrounding vessels and organs of 1 cm was maintained, even if this prevented complete tumor ablation.

The duodenum was perfused continuously with 100 ml cold saline per minute through one nasogastric tube and drained by a second, creating a cooling circulation to prevent thermal damage.

Bipolar RFA was executed using the CELON^®^ Power System generator (Olympus Surgical Technologies Europe, Teltow, Germany). A water-cooled RFA probe with active length of 20, 30, or 40 mm was used, depending on the size and shape of the tumor. The probe was placed in the lesion under ultrasonic guidance. Power was set to 1 W for every millimetre length of the active probe, causing tumoricidal temperature (90 °C). The ablation was ended when a total energy of 15 kJ per probe had been administered. Depending on tumor size, 1–4 probes were used.

In case of jaundice or expected biliary/gastric outlet obstruction, palliative bypass surgery was performed after RFA.

### CT-imaging pre- and post-RFA

CT-scans were performed at 1 week and 3 months after RFA in all patients according to protocol as standard regimen. If necessary for clinical reasons, additional imaging was done. CT-scans were performed according to the portal venous or biphasic protocol. Before the start, a region of interest was identified in the aorta and the threshold was set at 100 Hounsfield Unit (HU). Then, 125 ml intravenous contrast and 50 ml NaCl were injected at 3 and 5 ml/s, respectively.

For the portal venous scans, scanning was performed after a post-threshold delay of 49 s. The imaging was acquired cranial to caudal, with 0.9 mm slice thickness, 0.7 mm increment, 120 kV, and 128 mAs. Transversal, coronal, and sagittal reconstructions were made with a reconstruction interval of 1.25 mm.

For biphasic imaging, the arterial phase was acquired after a post-threshold delay of 20 s, from the lower edge of the diaphragm to the iliac crest. The full abdominal portal phase was made after a post-threshold delay of 55 s. The following parameters were used: 0.9 mm slice thickness, 0.7 mm increment, 120 kV, and 150 (arterial) or 300 (portal) mAs. A reconstruction interval of 5/4 mm was used for the coronal reconstruction and a 3/3 mm interval was used for the transversal and sagittal reconstructions.

### Data analysis

All pre- and 1 week and 3 months post-RFA CT-scans were collected. Two dedicated abdominal radiologists assessed all included scans in unison. Discordant judgments were addressed by discussion and consensus. The evaluation was done using standardized radiological scoring lists (Online Appendix 1). The main items of the list used for the pre-scans were tumor characteristics and vascular involvement.

For the scans post-RFA, ablation zone characteristics and vascular involvement in the ablation zone were added to the above-mentioned items. Furthermore, changes during follow-up in these characteristics were recorded.

Scan quality was defined as satisfactory, suboptimal (distorting factors, but still diagnostic) or non-diagnostic.

Viable tumor after ablation was defined as visible enhancement, mass effect, and progression over time. Tumor boundaries were considered well-defined or partially well-defined, depending on whether the whole circumference was clearly identifiable or only partially. If no clear distinction between tumor and surrounding tissue could be made, the tumor was considered ill-defined. ‘Vascular involvement’ is defined as any degree of circumferential involvement with arterial or venous structures. A lesion was considered heterogeneous if the highest and lowest HU-value differed more than 20 and/or if there were multiple content types (e.g., soft tissue and fluid). Content-type definition was: air if HU was < − 900, fluid if HU 0–20 and soft tissue if HU > 20. The definition of “ablation zone” is the lesion visible after RFA treatment.

### Statistics

Quantitative data were described with the mean and standard deviation (SD) in case of normal distribution. In case of non-normal distribution, median and interquartile ranges (IQR) were reported. Categorical data were described by frequency distribution with percentage. All statistical analyses were performed with a statistical software package (SPSS, version 21.0, Armonk, New York: International Business Machines Corporation).

## Results

All 18 consecutive patients were included in this radiological study, consisting predominantly of females (61%), with a median age of 64 years (IQR 52–70). 14/18 patients received no chemotherapy before or after RFA (Table 1). None of the patients received pre- or post-RFA radiation. RFA-related morbidity was 11% (2/18): one portal vein thrombosis and one duodenal injury occurred due to the procedure.

**Table 1:** Characteristics

	All patients (*n* = 18)
Patient characteristics	
Female	11 (61%)
Age (median [IQR])^a^	64 (52–70)
Induction chemotherapy	
None	16 (89%)
FOLFIRINOX	2 (11%)
Chemotherapy after RFA	
None	14 (78%)
FOLFIRINOX	1 (5.6%)
Gemcitabine	2 (11%)
FOLFIRINOX + gemcitabine	1 (5.6%)
Tumor characteristics	
Localization	
Head	14 (78%)
Corpus	4 (22%)
Largest diameter in mm (mean [SD])	
Transversal	44 (11)
Coronal	44 (10)
Vascular involvement	
SMA	13 (72%)
Celiac trunk	8 (44%)
CHA	12 (67%)
PV	13 (72%)
SMV	17 (94%)

^a^At RFA procedure; IQR, inter-quartile range; SMA, superior mesenteric artery; CHA, common hepatic artery; PV, portal vein; SMV, superior mesenteric vein

51 CT-scans were assessed (of which 47 (92%) biphasic). Pre-RFA and protocolled 1-week post-RFA scans were performed in all 18 patients. At 3 months of post-RFA, scans were conducted in 15 patients, since three patients had a postoperative survival of less than 3 months. The median survival in the total patient group was 8.8 months (range 2–21 months after RFA). All scans were sufficient for assessment, of which 1 (2%) was considered suboptimal but diagnostic, due to artefacts.

The tumor was located in the pancreatic head in 14 patients (78%) and the mean pre-RFA tumor size was 4.4 cm on transversal (SD 1.0) and coronal (SD 1.1) measurement. Pre-treatment, the SMV was involved in the tumor in 94% of patients and arterial involvement was present in 72% of patients (Table 1). A median of 2 probes (range 1–4) was used for the RFA procedure and palliative bypass surgery was frequently performed (hepaticojejunostomy 22%, gastrojejunostomy 6%, or both (double bypass) 44%).

### Ablation zone

Characteristics of the ablation zone are described in detail in Table 2.

**Table 2:** Ablation zone characteristics

Time after RFA (number of scans)	1 week (*n* = 18)	3 months (*n* = 15^a^)
Visible ablation zone	18 (100%)	5 (33%)
Location relative to tumor		
Central	2 (11%)	
Eccentric^b^	16 (89%)	
Attenuation^c^		
Homogeneous		
Soft tissue	2 (11%)	0
Fluid	0	2 (40%)
Heterogeneous		
Soft tissue	7 (39%)	1 (20%)
Fluid + soft tissue	8 (44%)	2 (40%)
Air + fluid + soft tissue	1 (6%)	0
Well-defined boundary^a^		
Yes (entire circumference)	11 (61%)	1 (20%)
Partially	4 (22%)	2 (40%)
No	3 (17%)	2 (40%)
Vascular involvement*		
SMA	4 (22%)	1 (20%)
Celiac trunk	2 (11%)	1(20%)
CHA	2 (11%)	0
PV	5 (28%)	0
SMV	8 (44%)	1 (20%)

SMA, superior mesenteric artery; CHA, common hepatic artery; PV, portal vein; SMV, superior mesenteric vein

^a^Three patients deceased prior to the 3 months follow-up CT scan

^b^Eccentric; not surrounded by tumor on all sides

^c^All percentages in this table are based on the number of visible ablations

**Fig. 1:**
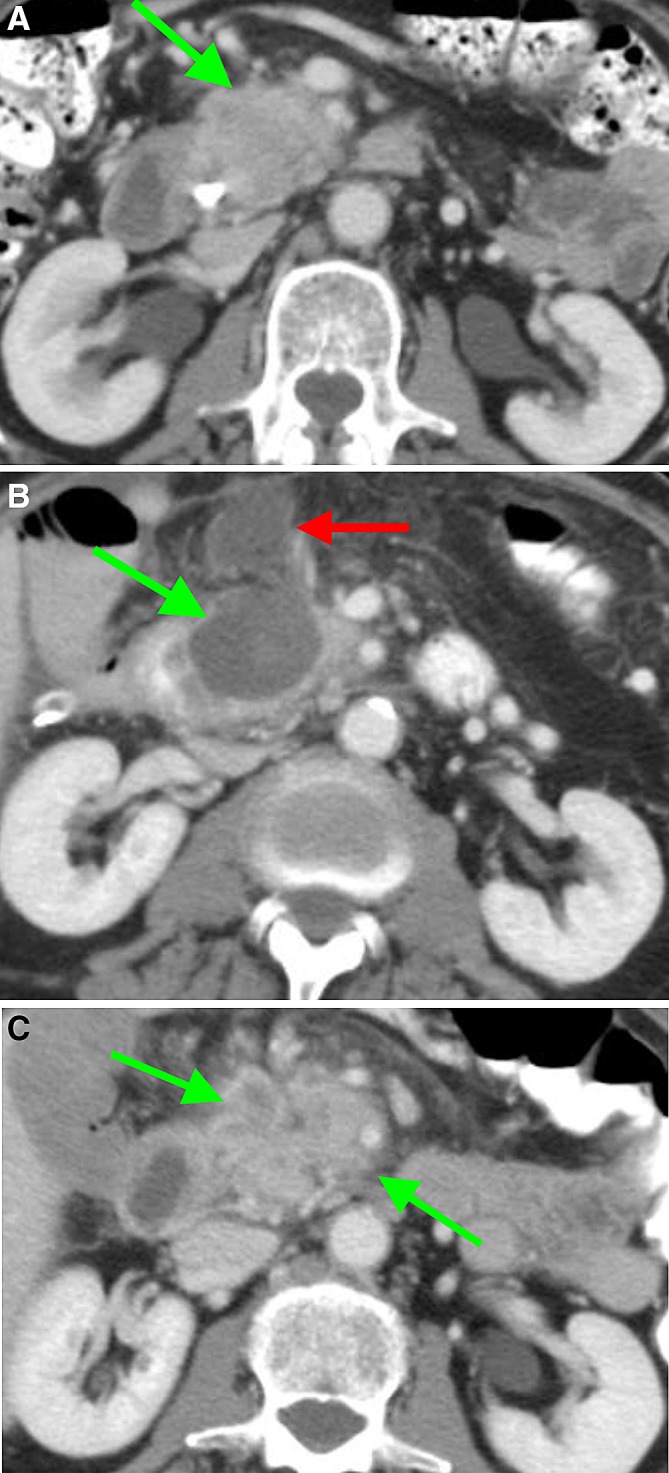
CT-images in the axial plane, of a 74-year-old woman with **A** a ductal adenocarcinoma in the head of the pancreas (arrow) on the pre-RFA scan, showing 90°–180° circumferential involvement of the superior mesenteric artery. **B** One-week post RFA, a sharply demarcated ablation zone is visible as a heterogeneous area (arrow). Moreover, the ablation zone extends ventrally outside the pancreas contour and a fluid collection is present in the transverse mesocolon. **C** Tumor replacing the ablation zone (arrow) at 3 months post-RFA. Note the inhomogeneous enhancement, the mass effect and the increasing size and involvement of the vessels over time

**Fig. 2:**
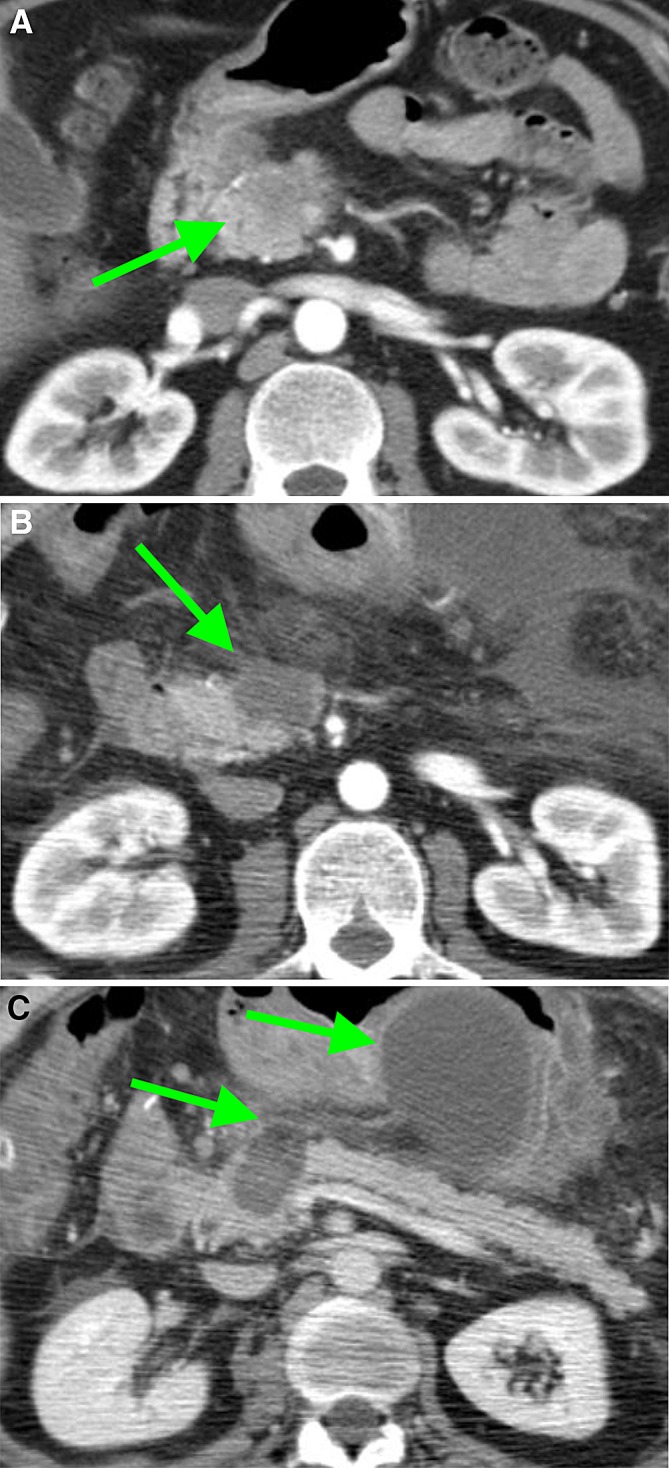
CT-images in the axial plane, of a 56-year-old man with **A** a ductal adenocarcinoma in the head of the pancreas (arrow) of 3.4 cm transversal on the pre-RFA scan. **B** and **C** On CT 1-week post-RFA, the ablation zone included the entire tumor (arrow). **C** The ablation zone included the ventral contour of the pancreas (left arrow) with an accompanying fluid collection in the lesser sac (right arrow)

One week after RFA, the ablation zone was visible in all patients, as a heterogeneous area in the majority of patients (89%), consisting of soft tissue and/or fluid components with various densities (Fig. 1A and B). The boundaries of the ablation zone were (partially) well-defined in 14 (83%) patients. In two patients (11%), the ablation zone included the entire tumor, including the vessels previously involved in the tumor (Fig. 2A and B). In the remaining patients, the ablation zone was positioned eccentric in relation to the tumor (89%), with viable tumor present on most, but not all borders of the ablation zone. In three patients, the ablation zone extended ventrally outside the pancreas, accompanied by fluid in the mesocolon and/or omental bursa.

At 3 months after RFA, in 10/15 patients (67%) the ablation zone was no longer visible on CT (Fig. 1C). Four of these patients had a 1-month scan, indicating the ablation zone disappeared between 1 and 3 months after RFA. Of the 5 remaining ablation zones, the boundaries became less distinct and the ablation zones were smaller compared to 1 week after RFA. Eight patients received additional CT-imaging at 6 months postoperatively, displaying only two remaining visible ablation zones. Three patients underwent CT at 9 months after RFA, showing no identifiable ablation zones, consistent with their previous scans.

Regarding the 2 patients, in whom the ablation zone included the entire tumor directly after RFA, tumor regrowth was already visible on the CT at 1 month post-RFA. Both patients deceased shortly after their CT at 3 months after RFA.

On the first CT-imaging after RFA, 21 vessels were involved in the ablation zone. In half of the patients, the SMV was involved, varying from < 90° to > 270°. Arterial involvement was more rare, predominantly affecting the SMA (22%) (Table 2 and Online Appendix 2).

**Fig. 3:**
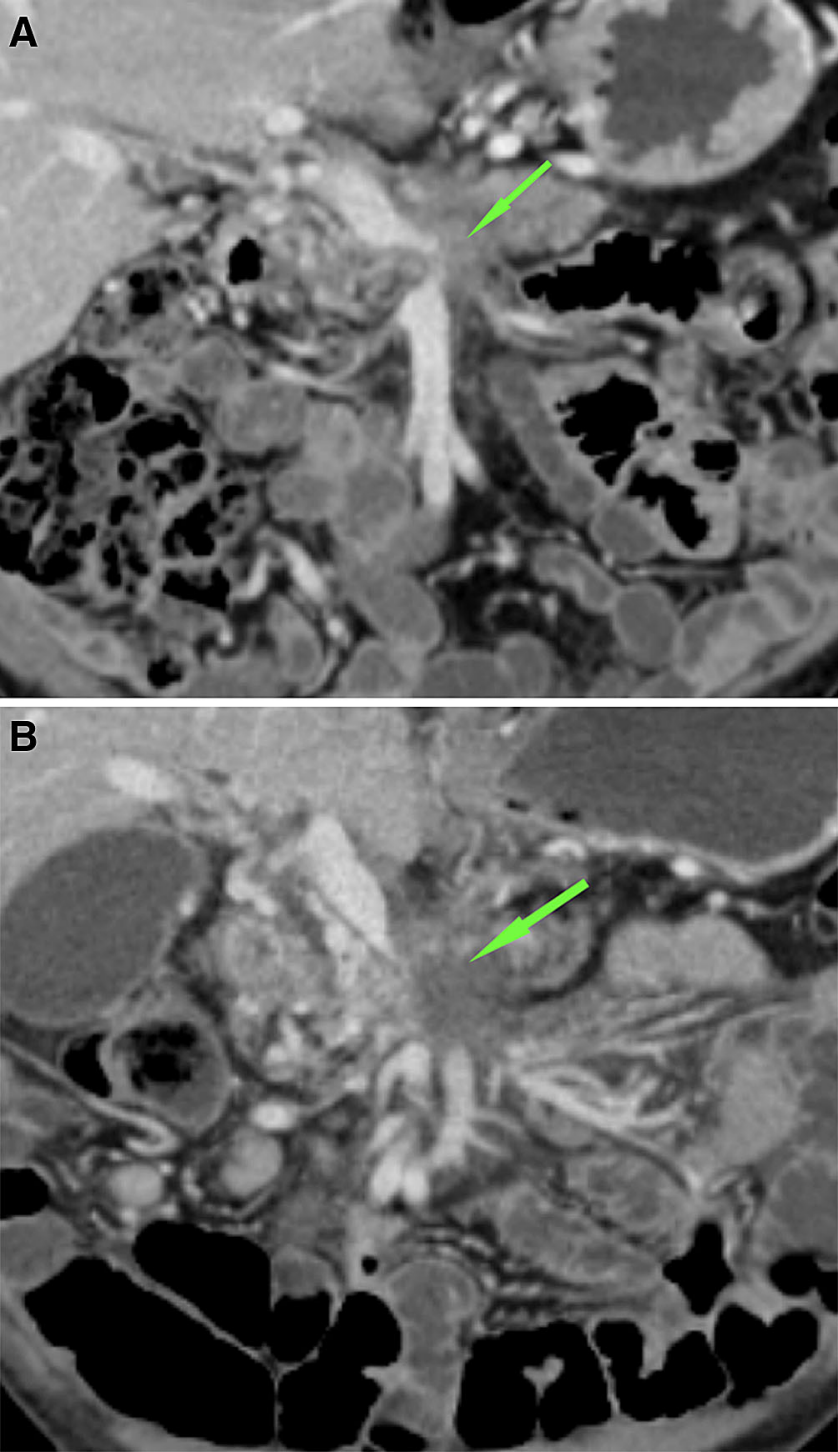
CT-images in the coronal plane, of the same patient as Fig. 6. **A** The superior mesenteric vein is already narrow on the pre-RFA imaging (arrow). **B** One week after RFA, the SMV is occluded for the part that is embedded in the ablation zone (arrow)

**Fig. 4:**
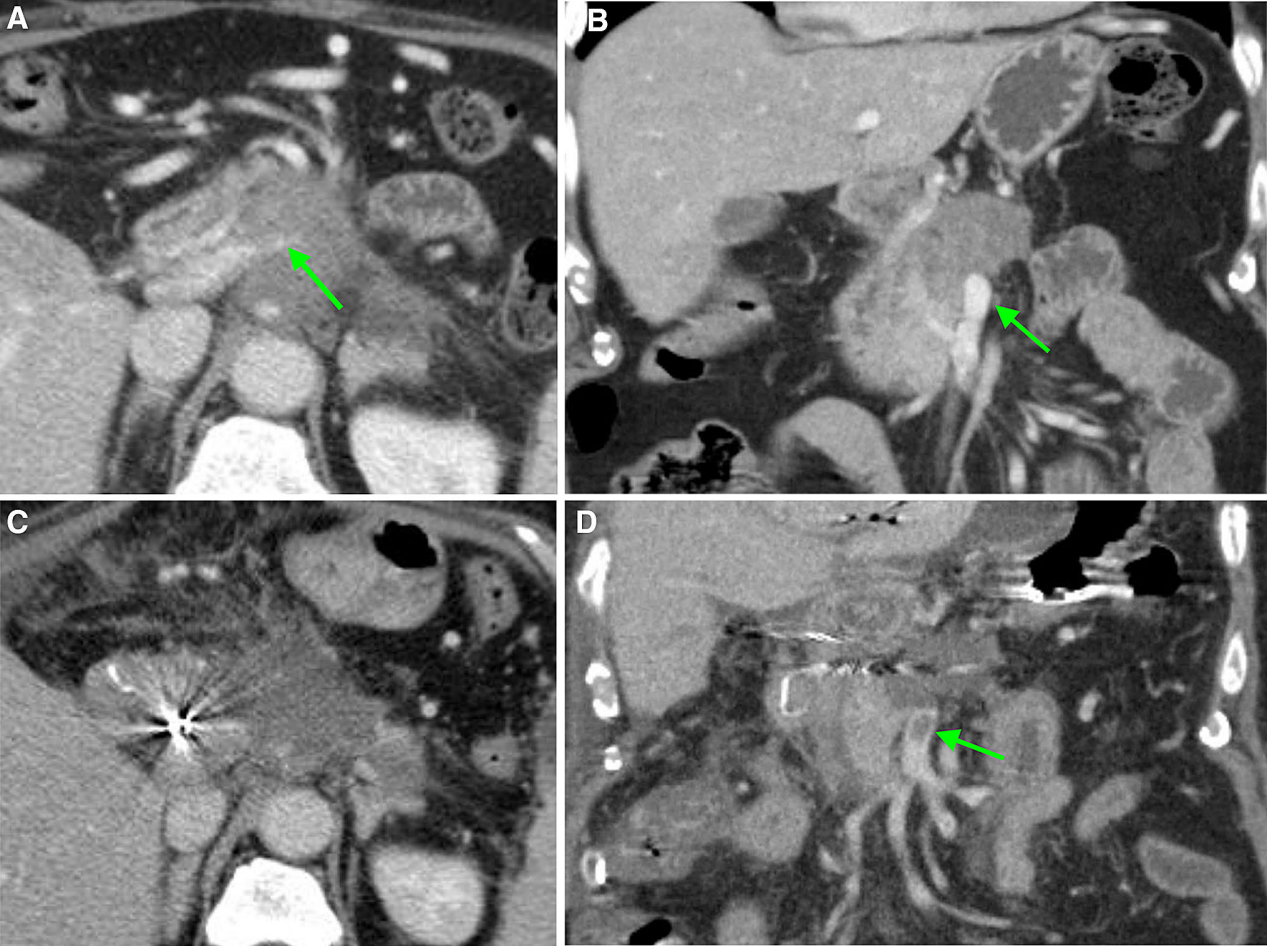
CT-images in the coronal plane, of a 66-year-old woman with a ductal adenocarcinoma in the head, neck and corpus of the pancreas. **A** The CT-image pre-RFA, shows a superior mesenteric vein (SMV) circumferential involvement of > 270°, with a pre-existent lumen reduction of > 50% (arrow) and **B** no thrombus present (arrow). **C** On the CT-image 1-week post-RFA the SMV and portal vein are occluded within the ablation zone and **D** a local thrombus is present in the SMV (arrow)

**Fig. 5:**
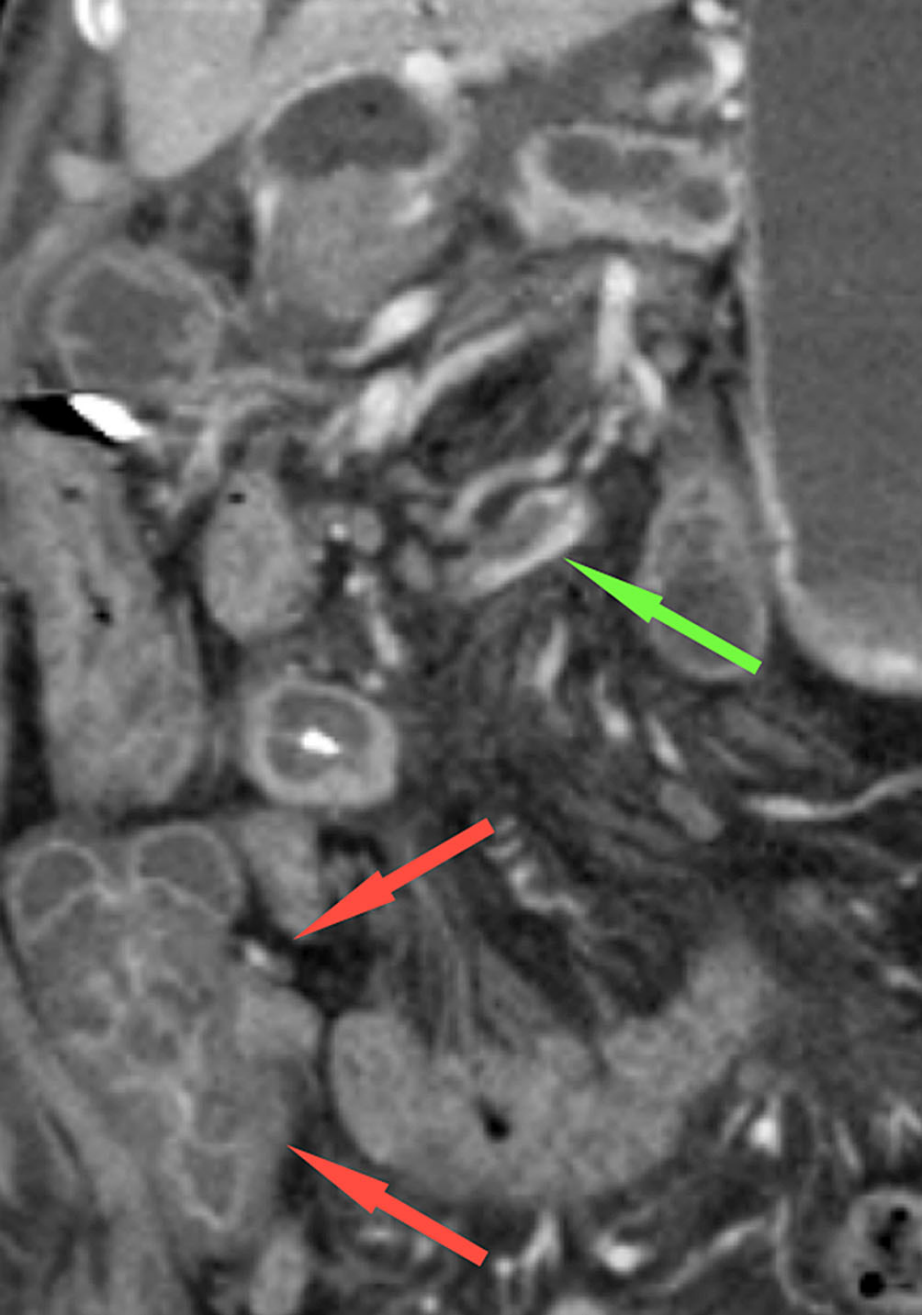
CT-image in the coronal plane, 1-week post-RFA, of a 68-year-old woman with a ductal adenocarcinoma located in the head and neck of the pancreas, showing a thrombus in the ileocolic vein (green arrow), resulting in edema of the ascending colon (red arrows)

Anatomical changes of vessels in contact with the ablation zone, such as thrombosis, shape distortion and occlusion, were mostly seen in venous structures (Table 3). In the portal vein (PV), lumen reduction occurred in 3 patients, of which two were partially involved in the ablation zone (< 180°) and partially in the tumor (< 270°). (Figure 3A and B) In the remaining case, the PV was entirely enclosed by the ablation zone, without remaining tumor contact. During follow-up, the vessels occluded, but at that point were only involved in the tumor, as no visible ablation zone remained. Furthermore, shape distortion was seen once, in case of a PV circumferential involvement > 270° in both the tumor and ablation zone. The distortion remained stable during follow-up. In two patients, the SMV was occluded 1 week after RFA, both being involved in the ablation zone for > 270°. In one of these vessels, a thrombus was present (Fig. 4A and B). The occlusions of the SMV persisted during follow-up (whilst the thrombus disappeared). One patient had asymptomatic edema of the ascending colon due to venous occlusion (Figure 5A and B).

**Table 3:** Ablation zone: vascular changes 1 week after RFA

Vessel involved in ablation zone	Lumen reduction	Thrombosis	Shape distortion	Occlusion
SMA (*n* = 4)	0	0	0	0
Celiac trunk (*n* = 2)	0	0	0	0
CHA (*n* = 2)	1	0	0	0
PV (*n* = 5)	3	0	1	0
SMV (*n* = 8)	0	1^a^	0	2^a^

SMA, superior mesenteric artery; CHA, common hepatic artery; PV, portal vein; SMV, superior mesenteric vein

^a^In one case, SMV with thrombus + occlusion

**Fig. 6:**
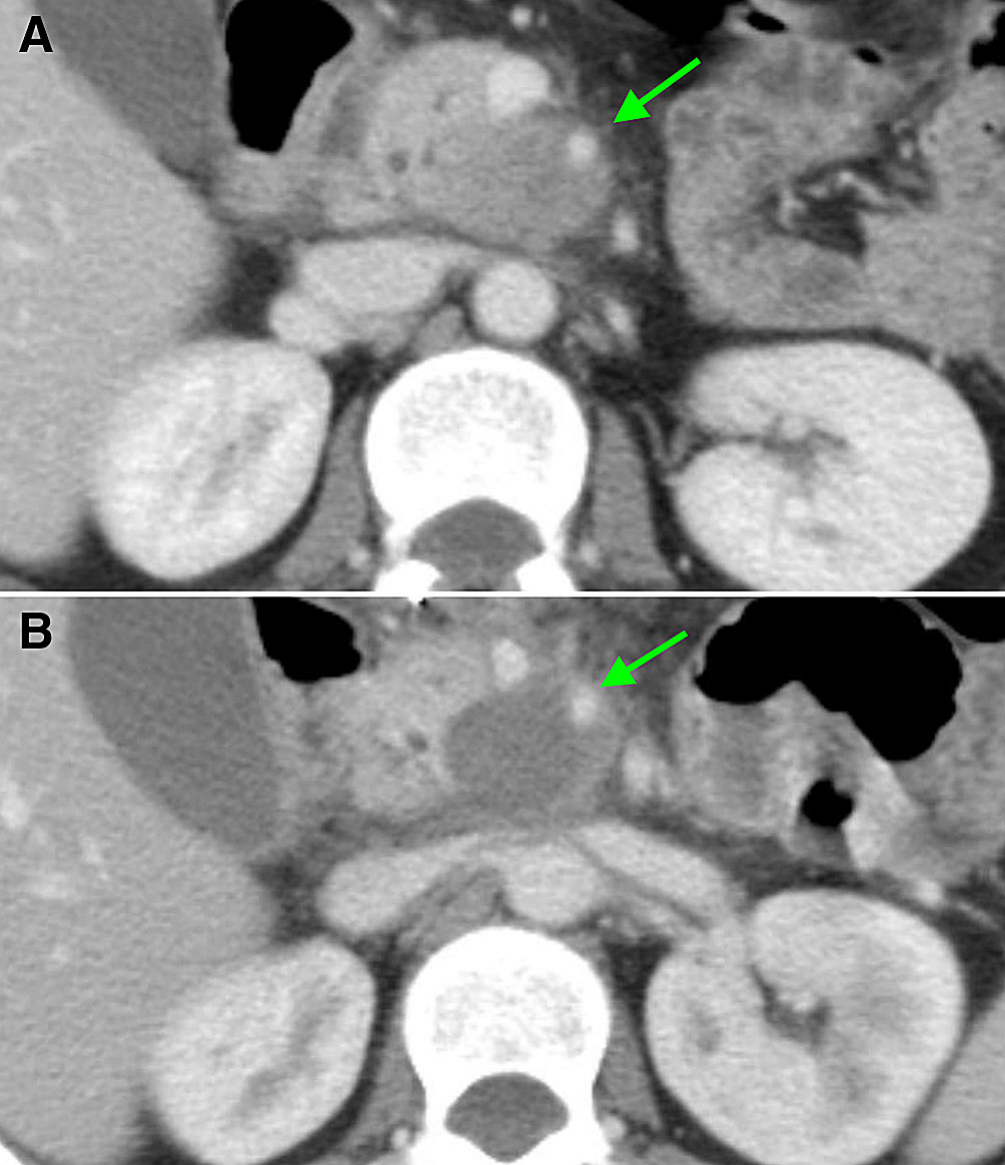
CT-images in the axial plane, of a 44-year-old woman with a ductal adenocarcinoma in the head of the pancreas. **A** On the pre-RFA CT-image, the superior mesenteric artery (SMA) was involved in the tumor, with a circumferential involvement of > 270, with normal patency and without anatomical changes. **B** On the CT-image 1-week post-RFA, the SMA was included in the ablation zone for 90°–180°, without the occurrence of anatomical changes

**Fig. 7:**
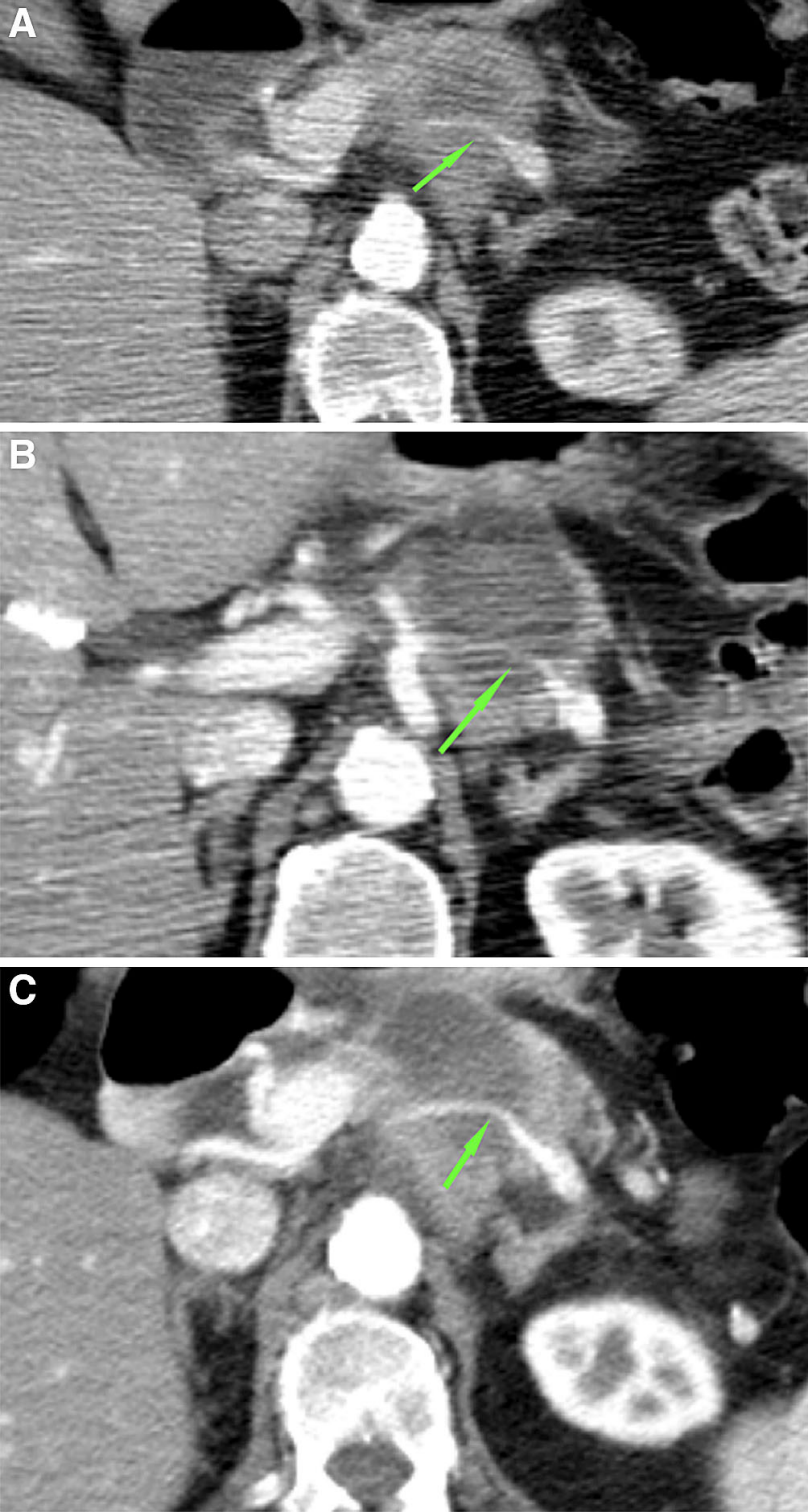
CT-images in the axial plane, of a 76-year-old woman with a ductal adenocarcinoma in the pancreatic corpus. **A** The splenic artery is encased by the tumor but still patent(arrow). **B** One week after RFA, the splenic artery is in contact with the ablation zone, but still patent (arrow). **C** This remained unchanged at follow-up 1 month later (arrow)

**Fig. 8:**
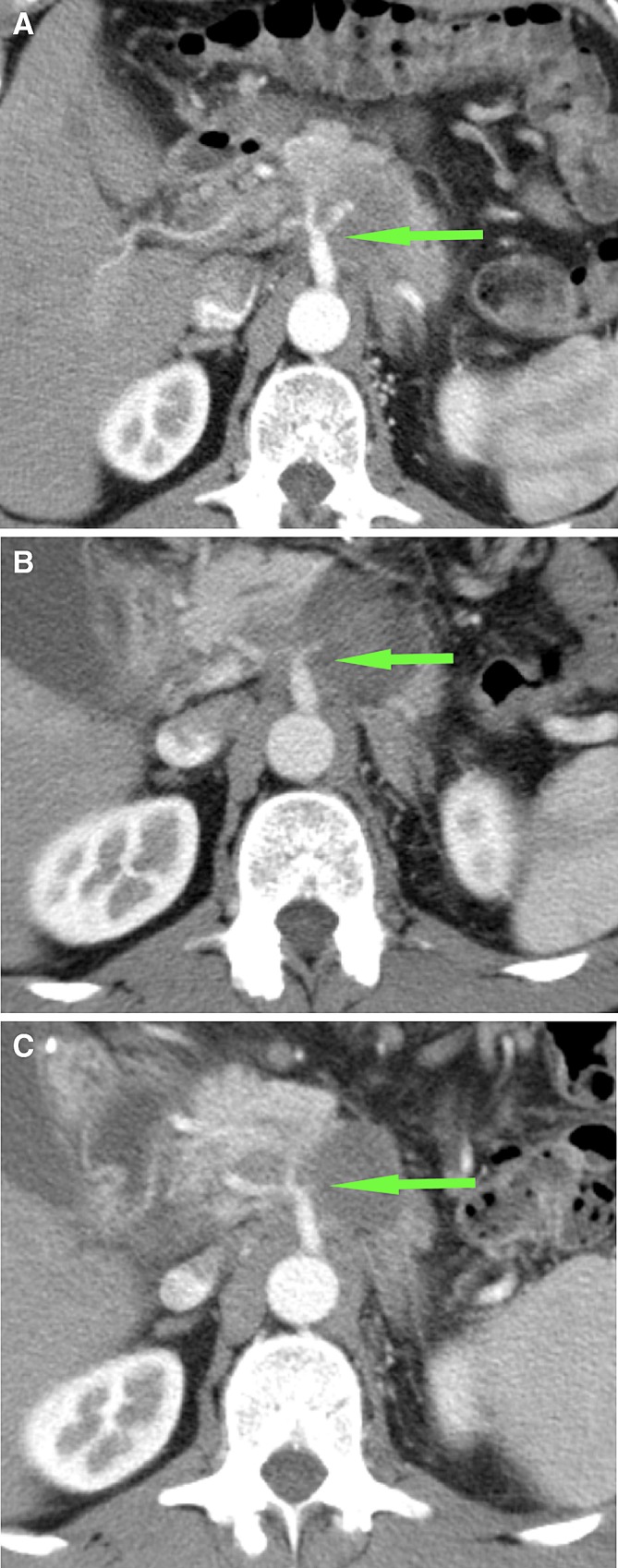
CT-images in the axial plane, of a 51-year-old man with a ductal adenocarcinoma in the corpus of the pancreas. **A** On the CT-imaging before RFA, the coeliac trunk and hepatic artery are closely related to the tumor. **B** One week after the procedure, these arteries are constricted but still patent (arrow). **C** This remained stable during follow-up 3 months later (arrow)

The SMA and celiac trunk involvement in the ablation zone never exceeded 180 degrees and no adverse effects were observed (Fig. 6). Also, no complications arose for the patient in whom the splenic artery was involved in the ablation zone (Fig 7A–C). In one case of complete tumor ablation, the CHA was constricted at 1-week post-RFA, where the contact with the ablation zone was < 180°. This remained stable during follow-up (Fig. 8A–C).

During follow-up, no new lumen reduction, thrombosis, or occlusion in previously unaffected vessels was seen.

The number of vessels involved in the remaining ablation zones decreased. On the CT-imaging performed 6 months after RFA (*n* = 8), no vessels were involved in the persisting ablation zones. (Table 2 and Online Appendix 2).

### Tumor characteristics during follow-up

Directly after RFA, no viable tumor was visible in two patients. In one other case, the bulk of the tumor was no longer visible due to the ablation, but tumor infiltration around the vascular structures was still present. For the remaining (83%) scans, the tumor was partially ablated. Tumor boundaries remained ill-defined (83%) and the tumor appeared mostly heterogeneous (67%), similar to the pre-RFA imaging (Table 4). One week after RFA, tumor diameter was decreased in 28%. Predominantly, tumor diameter increased (39%) or remained stable (22%), due to the enclosed ablation zone that attributed to measurement of the tumor diameter.

**Table 4:** Tumor characteristics

Time after RFA (number of scans)	Pre-RFA (*n* = 18)	1 week (*n* = 18)	3 months (*n* = 15^b^)
Visible tumor	18 (100%)	16 (89%)^**a**^	15 (100%)
Size in mm (mean [SD])			
Transversal	44 (11)	46 (12)	53 (16)
Coronal	44 (10)	52 (13)	54 (12)
Size change^c^			
Stable	NA	4 (22%)	2 (13%)
Decrease	NA	7 (39%)^d^	1 (7%)
Increase	NA	7 (39%)	12 (80%)
Attenuation^e^			
Homogeneous	0	3 (19%)^a,f^	2 (13%)^f^
Heterogeneous	18 (100%)	12 (75%)^a,f^	12 (80%)^f^
Well-defined boundary^e^			
Yes	3 (17%)^g^	1 (6%)^a^	0
No	15 (83%)	15 (94%)^a^	15 (100%)
Vascular involvement			
SMA	13 (72%)	13 (81%)^a^	13 (87%)
Celiac trunk	8 (44%)	9 (56%)^a^	8 (53%)
CHA	12 (67%)	10 (63%)^a^	10 (67%)
PV	13 (72%)	12 (75%)^a^	13 (87%)
SMV	17 (94%)	13 (81%)^a^	14 (93%)

NA, not applicable; SMA, superior mesenteric artery, CHA, common hepatic artery, PV, portal vein, SMV, superior mesenteric vein

^a^These percentages are based on the number of visible tumors

^b^Three patients deceased prior to the 3 months follow-up CT scan

^c^Change in diameter compared to the previously performed scan

^d^Including the two cases where no remaining tumor was visible

^e^Only applicable for cases with visible tumor

^f^Could not be evaluated in 1 scan due to artefacts

On CT-imaging one week after RFA, the number of vessels and degree of circumferential tumor involvement was decreased, particularly SMV involvement (Table 4). This was linked to the appearance of the ablation zone, with consequent translation of tumor involvement into ablation zone involvement. In addition, some vessels were involved in both the tumor and the ablation zone. The later scans showed a gradual increase in the amount of vessels involved per patient as well as in the degree of circumferential involvement seen. Consequently, venous (PV and/or SMV) constriction or occlusion was frequently seen at 3 months postoperatively (93%).

During follow-up, an increase in tumor diameter was predominantly seen (80% of patients at 3 months post-RFA).

### Other relevant findings

In three patients, in whom the ablation zone extended ventrally outside of the pancreas, a peripancreatic fluid collection was visible 1 week after RFA (Figs. 1B and 2C). This fluid regressed during follow-up in all three patients, without clinical intervention.

## Discussion

The main interest of this study was to evaluate the appearance of the ablation zone on CT, which was sharply defined 1 week after RFA for LAPC. In two patients, the tumor was no longer seen, suggesting complete ablation of the tumor. Three months after RFA, the ablation zone was no longer visible on CT-imaging in 67% of patients. Given the inhomogeneous enhancement of the replacing tissue, the obvious mass effect and the increasing size and circumferential vessel involvement over time, this appears consistent with tumor infiltration (Fig. 1C). Moreover, as the majority of patients (89%) had an incomplete ablation, this would confirm tumor infiltration even more. Additionally, tumor infiltration can also explain why the boundaries of the ablation zone became less defined over time. A potential other explanation is progression of necrosis and liquefaction of the ablated area [12]. However, no necrosis or liquefaction was identified on the CT-images in this study by the radiologists. Furthermore, shrinkage of the ablation zone, due to amelioration of the inflammatory reaction provoked by RFA, may also decrease the diameter of the ablation zone. If the associated oedema decreases, a smaller area remains, where the oedema shortly after RFA could have been mistaken for a part of the ablation zone [13–16]. Notably, we did not have pathological proof to support one of these explanations.

It would be of interest to explore the consequences of this incomplete ablation, by correlating the ablation volume to prognosis in future studies.

In three patients, the ablation zone extended ventrally outside of the pancreas, with a fluid collection visible in all three, suggesting leakage of pancreatic fluid. However, clinical intervention was not necessary and the collections regressed spontaneously. Nevertheless, one should be cautious with ablating up to the anatomical outline of the pancreas.

Regarding the vascular relation with the ablation zone, we demonstrated that vessel involvement was not uncommon. Despite the safety margin between duodenum and major vascular structures and the RFA probe of at least 1 cm, CT-imaging 1 week after RFA showed that 21 vessels were partly or fully enclosed in the ablation zone. The 1 cm distance was based on previously published experimental studies [17]. However, our results suggest that the maintained margin was not sufficient to preserve vessels from being involved in the ablated area, assuming that the margin was applied correctly. However, the unexpected high percentage of vessels involved in the ablation zone only led to venous thrombosis and/or occlusion in a minority of patients without clinical consequences and was without adverse effects in the patients of arterial involvement. Therefore, the presence of vessels in the ablated area should not be a contraindication for performing RFA.

Furthermore, it is not possible to fully discern the effects of RFA from the effects of tumor infiltration. An anatomical change (lumen reduction, shape distortion, and/or thrombosis) occurred in six venous vessels (SMV or PV) that were involved in the ablation zone, in four patients. However, in half of these patients, the affected vessels were partially involved in the ablation zone and partially involved in the tumor. In contrast, the one artery with lumen reduction had < 180° involvement in the ablation zone and no remaining tumor contact.

A limitation of this study is that it consists of a small, heterogeneous group of patients. In addition, there was a wide range in the post-RFA survival. This can be explained by the variance in additional therapy that the patients received and the fact that the pre-existing co-morbidity of the patients varied widely. These limitations have to be kept in mind when considering the survival data. However, this study was meant as a descriptive study and is the first to offer a systematically, detailed insight in the radiological findings directly after RFA and during follow-up.

Another limitation comprises the measurement of the tumor. It would be of interest to measure the volume of the tumor instead of the largest diameter. Given the ill-defined borders of the tumor, due to the infiltrative character of PDAC, resulting in ambiguous margins this could not be reliably assessed. Therefore, the maximal diameter was used to measure tumor size, as the most unambiguous method. Given that the largest tumor diameter often comprised (a part of) the ablation zone this measure does not accurately reflect the actual tumor volume after RFA.

In addition, pathological data to correlate to the CT findings were unavailable as none of the patients were found to be resectable after RFA treatment and exploration would therefore be unethical.

Lastly, we have to consider the impact of the used imaging modality. CT is the primary modality used in our institution, and world wide, for the imaging of pancreatic carcinoma both before and after treatment. Also, CT is the preferred imaging method for the visualization of the vascular structures surrounding the pancreas and of perivascular tumor infiltration. More importantly, we did not know on beforehand what the effects of the radiofrequency ablation would be on both the tumor and the surrounding vessels.

However, MRI is more suited for the differentiation of soft tissues than CT. As we have now gained more knowledge in the CT-appearance of both the tumor and ablation zone after RFA, plus the expected effects on both arterial and venous structures, new questions arise. As mentioned before, these questions include, among others, the differentiation between vital tumor and tumor necrosis and the differentiation between ablation zone and tumor.

Consequently, the role of MRI for the evaluation of the effects of RFA is something we want to explore in future studies.

In conclusion, 1 week after RFA in LAPC an ablation zone is visible in all patients. It usually appears as a sharply defined, heterogeneous area and is obliterated at 3 months follow-up, possibly due to tumor growth. The ablation zone regularly included vascular structures, with asymptomatic venous occlusion or thrombosis in a minority of patients and without adverse events in case of arterial involvement.

## Electronic supplementary material

Below is the link to the electronic supplementary material.

Supplementary material 1 (DOCX 47 kb)

Supplementary material 2 (DOCX 20 kb)
