# Supplementary material for: Computed tomography findings after radiofrequency ablation in locally advanced pancreatic cancer
Source: Abdom Radiol (NY). 2018 Feb 28;43(10):2702–11. doi: 10.1007/s00261-018-1519-y (PMC6132871; doi:10.1007/s00261-018-1519-y)
Supplement: Supplementary file 1 — Supplementary material 1 (DOCX 47 kb) [file 261_2018_1519_MOESM1_ESM.docx]

**APPENDIX 1 – Radiological scoring lists**

Article: Computed tomography findings after radiofrequency ablation in locally advanced pancreatic cancer

Journal: Abdominal Radiology

Authors: Steffi JE Rombouts MD, PhD, Tyche C Derksen MD, Chung Y Nio MD, Richard van Hillegersberg MD, PhD, Hjalmar C van Santvoort MD, PhD, Marieke S Walma MD, Izaak Q Molenaar MD, PhD, Maarten S van Leeuwen MD, PhD.

Corresponding author: M.S van Leeuwen, MD, PhD, University Medical Center Utrecht Cancer Center, Utrecht; E-mail: [m.s.vanleeuwen@umcutrecht.nl](mailto:m.s.vanleeuwen@umcutrecht.nl)

**1. Checklist for the CT-scan prior to RFA**

**Patient information**

| Name |  | |
| --- | --- | --- |
| Date of birth |  | |
| Induction chemotherapy | - Yes - No | If yes, describe:   - Type of chemotherapy: - Number of treatments: |
| Bypass | - Yes - No | If yes, describe: |

**General scan remarks**

| Scan date | **[ ] [ ] [ ]** |
| --- | --- |
| Modality | CT / MRI |
| Quality | Sufficient / Doubtful / insufficient |
| Scan type | Abdomen / pancreas |
| Scan phase(s) | Venous / arterial / parenchyma l |
| Date of reassessment |  |
| Names of radiologists |  |

**Tumor**

| Visible? | - Yes - No | If yes, the tumor is:   - Homogeneous - Heterogeneous | If homogeneous:   - Describe density (HU value):   If heterogeneous, describe tissue type and HU value:   - Air - Soft tissue - Fluid < 20HU ; >20HU |
| --- | --- | --- | --- |
| Localization | - Head - Neck (ventral of confluence/ VMS) - Corpus - Tail | | |
| Size (in mm) | - Sagittal: - Coronal: - Transversal: | | |
| Well-defined boundary | - Yes - No - Partially | | |
| Invasion of surrounding structures | - Yes - No | If yes, describe invasion:   - Peripancreatic fat - Towards SMA - Transverse mesocolon - Mesenterial root - Towards Cava/Aorta - Cranial towards coeliac trunk - Dorsal of pancreatic corpus/tail - Other, describe: - Duodenum - Stomach - Hepatoduodenal ligament - Jejunum - Colon - Left adrenal gland - Spleen - Other, describe: | |

**Vascular involvement**

| Arterial anatomy | - Normal - Aberrant | | If aberrant, describe:   - Accessory branch: replaced LHA from LGA - Accessory branch: replaced RHA from SMA - Replaced CHA from SMA - Early division of CHA with posterior position of RHA - Other, describe: | | |
| --- | --- | --- | --- | --- | --- |
| SMA | Patency | | - Normal - Stenosis | | |
|  | - Involvement (degrees) - No involvement | | - < 90 - 90-180 - 180-270 - >270 - 360 | | |
|  | Lumen reduction | | - No - <50% - >50% - Occlusion | | |
|  | Thrombus present | | - Ýes - No | | |
| Coeliac trunk | Patency | | - Normal - Stenosis: A) arcuate ligament B) atherosclerosis | | |
|  | - Involvement (degrees) - No involvement | | - < 90 - 90-180 - 180-270 - >270 - 360 | | |
|  | Lumen reduction | | - No - <50% - >50% - Occlusion | | |
|  | Thrombus present | | - Yes - No | | |
| CHA | - Involvement (degrees) - No involvement | | - < 90 - 90-180 - 180-270 - >270 - 360 | | |
|  | Lumen reduction | | - No - <50% - >50% - Occlusion | | |
|  | Thrombus present | | - Yes - No | | |
| VP | - Involvement (degrees) - No involvement | | - < 90 - 90-180 - 180-270 - >270 - 360 | | |
|  | Length of contact (in mm): | | | Including SMV length: yes / no | |
|  | Shape distortion | | - Yes - No | | |
|  | Lumen reduction | | - No - <50% - >50% - Occlusion | | |
|  | Thrombus in vat | | - Yes - No | | |
| SMV | - Involvement (degrees) - No involvement | - < 90 - 90-180 - 180-270 - >270 - 360 | | | |
|  | Length of contact (in mm): | | | | Including VP length: yes / no |
|  | Shape distortion | - Yes - No | | | |
|  | Lumen reduction | - No - <50% - >50% - Occlusion | | | |
|  | Thrombus in vat | - Yes - No | | | |
| Collaterals visible | - Yes - No | If yes, describe:   - Arterial, localization:…………………………………………….. - Venous, localization:……………………………………………... | | | |

**Parenchymal condition**

| - Normal - Aberrant | If aberrant:   - Atrophic - Pancreatitis component - Cysts - Other, describe: |
| --- | --- |

**Lymph nodes**

| Suspect lymph nodes  (short axis ≥10mm or <10mm but spherical, hypodens or ill-defined boundary) | - Yes - No | If yes, describe location:   - Regional (location + amount): - Distant (location + amount): |
| --- | --- | --- |

**Metastases**

| - Yes - No | If yes, describe:   - Location: - Amount: |
| --- | --- |

**Other**

| Radiologic TNM classification | - T - N - M | |
| --- | --- | --- |
| Stent in situ | - Yes - No | - Metal - Plastic |
| Max diameter CHD/CBD (in mm): | | |
| Max diameter pancreatic duct (in mm): | | |
| Relevant other findings:   - Cyst(s) - Ascites - Abscess(es) - Other, describe: | | |

**2. Checklist for the CT-scans after RFA**

**Patient information**

| Name |  | |
| --- | --- | --- |
| Date of birth |  | |
| Adjuvant chemotherapy | - Yes - No | If yes, describe:   - Type of chemotherapy: - Number of treatments: |
| Bypass | - Yes - No | If yes, describe: |

**General scan remarks**

| Scan date  Number of months post RFA | **[ ] [ ] [ ]**  **[ ]** Scan number 1 2 3 4 5 6 7 post RFA (encircle) |
| --- | --- |
| Modality | CT / MRI |
| Quality | Sufficient / Doubtful / insufficient |
| Scan type | Abdomen / pancreas |
| Scan phase(s) | Venous / arterial / parenchyma l |
| Date of reassessment |  |
| Names of radiologists |  |

**Tumor**

| Visible? | - Yes - No | If yes, the tumor is:   - Homogeneous - Heterogeneous | If homogeneous:   - Describe density (HU value):   If heterogeneous, describe tissue type and HU value:   - Air - Soft tissue - Fluid < 20HU ; >20HU |
| --- | --- | --- | --- |
| Localization | - Head - Neck (ventral of confluence/ VMS) - Corpus - Tail | | |
| Size (in mm) | - Sagittal: - Coronal: - Transversal: | | |
| Well-defined boundary | - Yes - No - Partially | | |
| Invasion of surrounding structures | - Yes - No | If yes, describe invasion:   - Peripancreatic fat - Towards SMA - Transverse mesocolon - Mesenterial root - Towards Cava/Aorta - Cranial towards coeliac trunk - Dorsal of pancreatic corpus/tail - Other, describe: - Duodenum - Stomach - Hepatoduodenal ligament - Jejunum - Colon - Left adrenal gland - Spleen - Other, describe: | |

**Tumor**

| Change in tumor size | - Yes - No | If yes, describe: |
| --- | --- | --- |
| Tumor shape distortion? | - Yes - No | If yes, describe: |
| Change in tumor invasion of surrounding structures | - Ja - Nee | If yes, describe: |

**Vascular involvement**

| SMA | - Involvement (degrees) - No involvement | - < 90 - 90-180 - 180-270 - >270 - 360 | |
| --- | --- | --- | --- |
|  | Lumen reduction | - No - <50% - >50% - Occlusion | |
|  | Thrombus present | - Ýes - No | |
|  | Changed compared to previous scan? | - Yes - No | If yes, describe: |
| Coeliac trunk | - Involvement (degrees) - No involvement | - < 90 - 90-180 - 180-270 - >270 - 360 | |
|  | Lumen reduction | - No - <50% - >50% - Occlusion | |
|  | Thrombus present | - Ýes - No | |
|  | Changed compared to previous scan? | - Yes - No | If yes, describe: |
| CHA | - Involvement (degrees) - No involvement | - < 90 - 90-180 - 180-270 - >270 - 360 | |
|  | Lumen reduction | - No - <50% - >50% - Occlusion | |
|  | Thrombus present | - Yes - No | |
|  | Changed compared to previous scan? | - Yes - No | If yes, describe: |

| VP | - Involvement (degrees) - No involvement | - < 90 - 90-180 - 180-270 - >270 - 360 | | |
| --- | --- | --- | --- | --- |
|  | Length of contact (in mm): | | | Including SMV length: yes / no |
|  | Shape distortion | - Yes - No | | |
|  | Lumen reduction | - No - <50% - >50% - Occlusion | | |
|  | Thrombus in vat | - Yes - No | | |
|  | Changed compared to previous scan? | - Yes - No | If yes, describe: | |
| SMV | - Involvement (degrees) - No involvement | - < 90 - 90-180 - 180-270 - >270 - 360 | | |
|  | Length of contact (in mm): | | | Including VP length: yes / no |
|  | Shape distortion | - Yes - No | | |
|  | Lumen reduction | - No - <50% - >50% - Occlusion | | |
|  | Thrombus in vat | - Yes - No | | |
|  | Changed compared to previous scan? | - Yes - No | If yes, describe: | |
| Collaterals visible | - Yes - No | If yes, describe:   - Arterial, localization:…………………………………………….. - Venous, localization:……………………………………………... | | |
|  | Changed compared to previous scan? | - yes - No | If yes, describe: | |

**Ablation zone**

| Visible? | - Yes - No | If yes, the tumor is:   - Homogeneous - Heterogeneous | | If homogeneous:   - Describe density (HU value):   If heterogeneous, describe tissue type and HU value:   - Air - Soft tissue - Fluid < 20HU ; >20HU |
| --- | --- | --- | --- | --- |
| Position relative to the tumor | - In the centre - Eccentric; describe involved sides/quadrants: | | | |
| Size (in mm) | - Sagittal: - Coronal: - Transversal: | | | |
| Well-defined boundary | - Yes - No - Partially | | | |
| Changed compared to previous scan? | - Yes - No - Not applicable (n.a.) * | | If yes, describe: | |

* if concerning first scan post RFA

**Relation of ablation zone with surrounding vessels**

| AMS | - Involvement (degrees) - No involvement | - < 90 - 90-180 - 180-270 - >270 - Anatomic changes (pseudo-aneurysm, thrombosis, shape distortion, lumen reduction, etcetera): | |
| --- | --- | --- | --- |
| Changed compared to previous scan? | | - Yes - No - N.a. * | If yes, describe: |
| Coeliac trunk | - Involvement (degrees) - No involvement | - < 90 - 90-180 - 180-270 - >270 - Anatomic changes (pseudo-aneurysm, thrombosis, shape distortion, lumen reduction, etcetera): | |
| Changed compared to previous scan? | | - Yes - No - N.a. * | If yes, describe: |
| AHC | - Involvement (degrees) - No involvement | - < 90 - 90-180 - 180-270 - >270 - Anatomic changes (pseudo-aneurysm, thrombosis, shape distortion, lumen reduction, etcetera): | |
| Changed compared to previous scan? | | - Yes - No - N.a. * | If yes, describe: |
| VP | - Involvement (degrees) - No involvement | - < 90 - 90-180 - 180-270 - >270 - Anatomic changes (pseudo-aneurysm, thrombosis, shape distortion, lumen reduction, etcetera): | |
| Changed compared to previous scan? | | - Yes - No - N.a. * | If yes, describe: |
| SMV | - Involvement (degrees) - No involvement | - < 90 - 90-180 - 180-270 - >270 - Anatomic changes (pseudo-aneurysm, thrombosis, shape distortion, lumen reduction, etcetera): | |
| Changed compared to previous scan? | | - Yes - No - N.a. * | If yes, describe: |

* if concerning first scan post RFA

**Parenchymal condition**

| - Normal - Aberrant | If aberrant:   - Atrophic - Pancreatitis component - Cysts - Other, describe: | |
| --- | --- | --- |
| Changed compared to previous scan? | - Yes - No | If yes, describe: |

**Lymph nodes**

| Suspect lymph nodes  (short axis ≥10mm or <10mm but spherical, hypodens or ill-defined boundary) | - Yes - No | If yes, describe location:   - Regional (location + amount): - Distant (location + amount): |
| --- | --- | --- |
| Changed compared to previous scan? | - Yes - No | If yes, describe: |

**Metastases**

| - Yes - No | If yes, describe:   - Location: - Amount: | |
| --- | --- | --- |
| Changed compared to previous scan? | - Yes - No | If yes, describe: |

**Other**

| Radiologic TNM classification | - T - N - M | |
| --- | --- | --- |
| Stent in situ | - Yes - No | - Metal - Plastic |
| Max diameter CHD/CBD (in mm): | | |
| Max diameter pancreatic duct (in mm): | | |
| Changed compared to previous scan? | - Yes - No | If yes, describe: |
| Relevant other findings | Change in previous findings:   - Yes - No | If yes, describe the changes: |
|  | New relevant findings?   - Yes - No | If yes, describe: |
