# Supplementary material for: Computed tomography findings after radiofrequency ablation in locally advanced pancreatic cancer
Source: Abdom Radiol (NY). 2018 Feb 28;43(10):2702–11. doi: 10.1007/s00261-018-1519-y (PMC6132871; doi:10.1007/s00261-018-1519-y)
Supplement: Supplementary file 2 — Supplementary material 2 (DOCX 20 kb) [file 261_2018_1519_MOESM2_ESM.docx]

**APPENDIX 2 – Vascular involvement in ablation zone and tumor**

Article: Computed tomography findings after radiofrequency ablation in locally advanced pancreatic cancer

Journal: Abdominal Radiology

Authors: Steffi JE Rombouts MD, PhD, Tyche C Derksen MD, Chung Y Nio MD, Richard van Hillegersberg MD, PhD, Hjalmar C van Santvoort MD, PhD, Marieke S Walma MD, Izaak Q Molenaar MD, PhD, Maarten S van Leeuwen MD, PhD.

Corresponding author: M.S van Leeuwen, MD, PhD, University Medical Center Utrecht Cancer Center, Utrecht; E-mail: [m.s.vanleeuwen@umcutrecht.nl](mailto:m.s.vanleeuwen@umcutrecht.nl)

| **Table. Ablation zone: vascular involvement** | | | |
| --- | --- | --- | --- |
| Time after RFA (N scans) | | 1 week (n=18) | 3 months (n=15) |
| Visible ablation zone | | 18 (100%) | 5 (33%) |
| SMA | <90 | 3 | 1 |
|  | 90-180 | 1 | - |
|  | 180-270 | - | - |
|  | >270 | - | - |
| Coeliac trunk | <90 | 2 | 1 |
|  | 90-180 | - | - |
|  | 180-270 | - | - |
|  | >270 | - | - |
| CHA | <90 | - | - |
|  | 90-180 | 1 | - |
|  | 180-270 | - | - |
|  | >270 | 1 | - |
| PV | <90 | 1 | - |
|  | 90-180 | 2 | - |
|  | 180-270 | - | - |
|  | >270 | 2 | - |
| SMV | <90 | 1 | - |
|  | 90-180 | 4 | - |
|  | 180-270 | - | - |
|  | >270 | 3 | 1 |
| SMA superior mesenteric artery, CHA common hepatic artery, PV portal vein, SMV superior mesenteric vein; NA not applicable | | | |

| **Table. Tumor: vascular involvement** | | | |
| --- | --- | --- | --- |
| Time after RFA (N scans) | | 1 week (n=18) | 3 months (n=15) |
| SMA | <90 | 2 | 2 |
|  | 90-180 | 1 | - |
|  | 180-270 | 1 | 1 |
|  | >270 | 9 | 10 |
| Celiac trunk | <90 | 2 | - |
|  | 90-180 | - | - |
|  | 180-270 | - | 2 |
|  | >270 | 7 | 6 |
| CHA | <90 | - | 1 |
|  | 90-180 | 2 | 1 |
|  | 180-270 | 1 | 1 |
|  | >270 | 7 | 7 |
| PV | <90 | - | - |
|  | 90-180 | 2 | 2 |
|  | 180-270 | - | 2 |
|  | >270 | 10 | 9 |
| SMV | <90 | - | 1 |
|  | 90-180 | 3 | 1 |
|  | 180-270 | 2 | - |
|  | >270 | 8 | 12 |
| SMA superior mesenteric artery, CHA common hepatic artery, PV portal vein, SMV superior mesenteric vein | | | |
